# A versatile breast reduction technique: Conical plicated central U shaped (COPCUs) mammaplasty

**DOI:** 10.1186/1750-1164-3-7

**Published:** 2009-07-03

**Authors:** Eray Copcu

**Affiliations:** 1 Department of Plastic Reconstructive and Aesthetic Surgery, Medical Faculty, Adnan Menderes University, 09100, Aydin, Turkey

## Abstract

**Background:**

There have been numerous studies on reduction mammaplasty and its modifications in the literature. The multitude of modifications of reduction mammaplasty indicates that the ideal technique has yet to be found. There are four reasons for seeking the ideal technique. One reason is to preserve functional features of the breast: breastfeeding and arousal. Other reasons are to achieve the real geometric and aesthetic shape of the breast with the least scar and are to minimize complications of prior surgical techniques without causing an additional complication. Last reason is the limitation of the techniques described before. To these aims, we developed a new versatile reduction mammaplasty technique, which we called conical plicated central U shaped (COPCUs) mammaplasty.

**Methods:**

We performed central plication to achieve a juvenile look in the superior pole of the breast and to prevent postoperative pseudoptosis and used central U shaped flap to achieve maximum NAC safety and to preserve lactation and nipple sensation. The central U flap was 6 cm in width and the superior conical plication was performed with 2/0 PDS. Preoperative and postoperative standard measures of the breast including the superior pole fullness were compared.

**Results:**

Forty six patients were operated with the above mentioned technique. All of the patients were satisfied with functional and aesthetic results and none of them had major complications. There were no changes in the nipple innervation. Six patients becoming pregnant after surgery did not experience any problems with lactation. None of the patients required scar revision.

**Conclusion:**

Our technique is a versatile, safe, reliable technique which creates the least scar, avoids previously described disadvantages, provides maximum preservation of functions, can be employed in all breasts regardless of their sizes.

## Background

**Figure 1:**
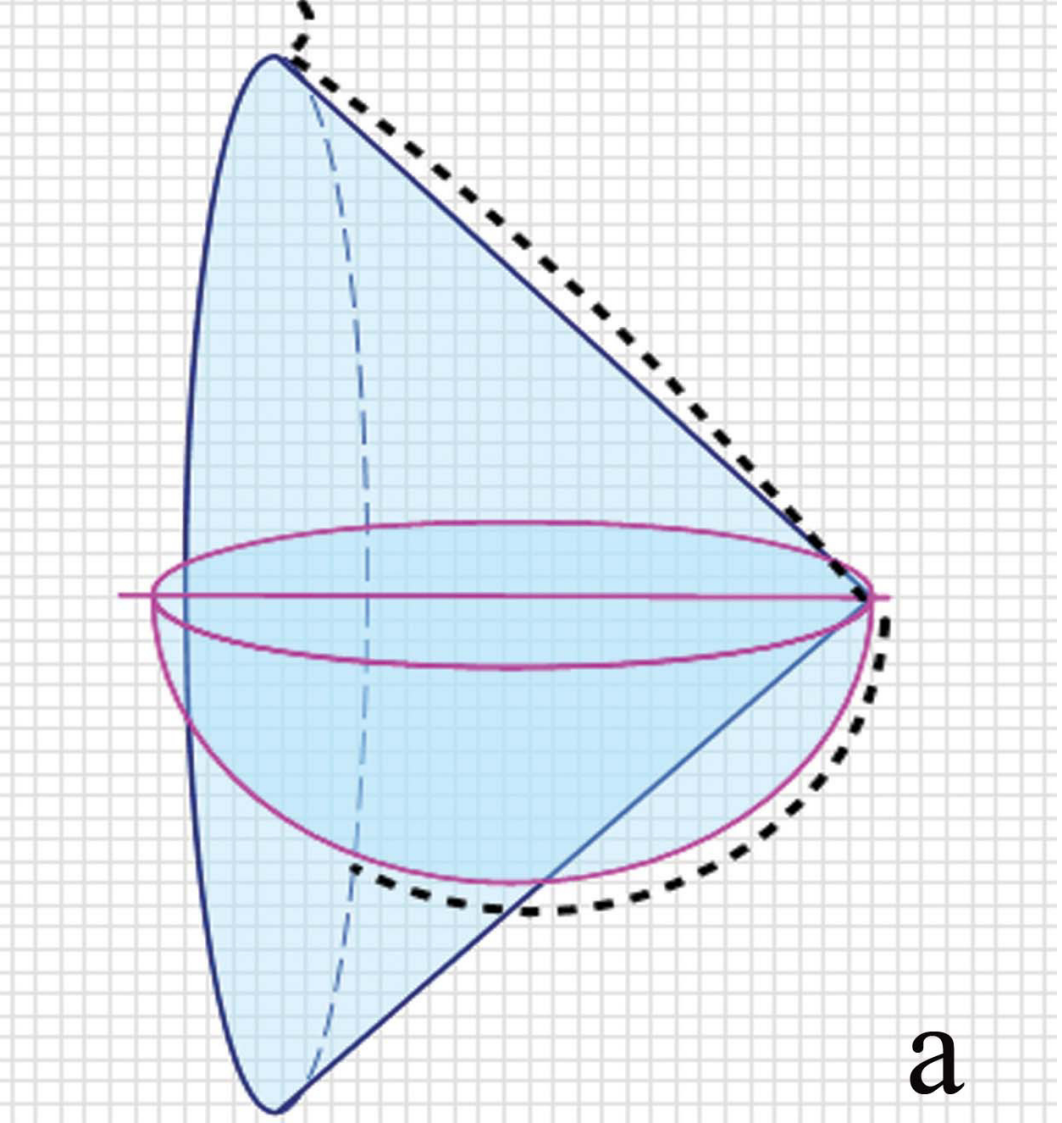
**Geometry of the breast**. Upper part of breast is half cone and lower pole is half a globe.

**Figure 2:**
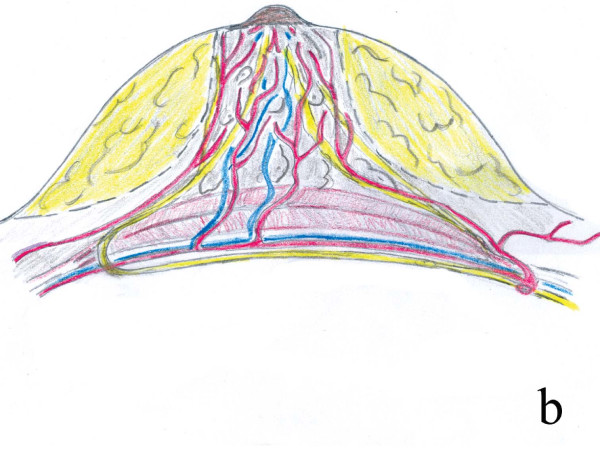
**Vascular, neural and glandular anatomy of the breast**. Dotted lines indicate the margins of the pedicle in our technique. Major vessels, nerves, and lactipherous ducts to the NAC and mammary gland are preserved in our technique.

The breast is one of the most important female organs. The breast has major implications in sexual arousal, as a result of its visual and sensual properties. Also most important feature of the breast is its capability of milk production. None of the plastic surgery operations put as much a heavy burden on plastic surgeons as reduction mammaplasty. Until today, many reduction mammaplasty techniques were described in the literature but the search for an ideal technique continues. In fact, surgical outcomes should not only fulfill patient expectations for an aesthetic appearance but also provide important breast functions. We believe that the most important philosophy in breast reduction surgery should be preservation of reliable neurovascular and lactational integrity to the nipple. Poor outcomes both affect the women undergoing operation and cause babies to feed on less breast milk, which is not acceptable. Attempts to find the ideal technique for reduction mammaplasty may end only if two secrets are resolved. One is to create the original geometry of the breast and the other is to provide maximum preservation of breast functions. The breast has a very complicated geometry. The complex geometry of the breast has been analysed with a three dimensional laser scanner in many studies [1]. The well-known definition of the breast is a cone horizontally projecting from the anterior thoracic wall [2]. Due to both effects of gravity and the nature of the breast tissue, the superior pole of the breast is a half a cone and the inferior pole is a half a globe (Figure 1). Andrades in a review on reduction mammaplasty techniques emphasized preservation and reconstruction of this cone shape [3]. Functional results of breast reduction are as important as its aesthetic results. The Surgeon General's health goals for 2010 are that 75% of woman initiates breastfeeding and that 50% continue it through 6 months postpartum [4]. Maximum preservation of breast functions depends on exact knowledge of anatomical features of the breast. At present, vascularization and innervation of the nipple areola complex (NAC) has been clearly described and the vessels and the nerves have been shown to reach vertically the NAC at the fourth and fifth ribs through a separate fibrous septum [5,6]. Although there is both deep and superficial blood supply system, generally accepted that vascularization and innervation of the NAC is through the central breast parenchyma which can be seen as inferior to the breast shape in standing position. Using central pedicle preserves nerve supply together with the vascular supply to the maximal extent possible in breast reduction surgery [7]. If the glandular tissue is not removed with the central pedicle, then the patient keeps her lactation potential with good nipple sensation [8]. The principle underlying the technique described here is complete preservation of these tissues (Figure 2).

A larger pedicle does not necessarily achieve better breast functions. Vessels and nerves of the NAC should be completely preserved. As a matter of fact, a large pedicle may cause such complications as displacement and folding of the flaps [9]. So that the breasts look natural after reduction mammaplasty, it can move to all directions and has a soft texture. It has been emphasized that a gland connected to the ducts and the nipple should be preserved for a successful breastfeeding following breast reduction [10]. However, to our knowledge, there have not been any studies showing how much breast tissue should be preserved for sufficient milk production. Maintenance of lactation should never be disregarded. Therefore, a maximum amount of the gland should be preserved. In all techniques except for the central or total posterior pedicle, the pedicle is not based on the gland only. In fact, most of the candidates for reduction mammaplasty have a high BMI[10]; that is, they have fat tissue as much as breast tissue.

Although vertical scar mammaplasty techniques are quite popular now, the most frequently performed technique is inferior pedicled mammaplasty. Critics cite a longer operating time than for vertical mammoplasties, the known scar problems of the Wise inverted T pattern, and the phenomenon of pseudoptosis, often termed "bottoming out"[11]. In our technique, conical plication creates fullness in the superior pole which in turn leads to a juvenile look, the technique does not cause postoperative pseudoptosis which frequently occurs in mammaplasty techniques, central U shaped pedicle allows maximum preservation of functions and the technique is applicable in all breasts irrespective of their sizes. The techniques described in the literature generally were not suitable for very big breasts but our approach is a versatile breast reduction technique. It can be used for all kind of breasts. There is no limitation to the amount of NAC repositioning that can be achieved. In short, maximum preservation of functions and an aesthetic breast with minimum scar are the main goals of the COPCUs mammaplasty.

## Methods

This technique was a modification of the total posterior pedicled mammaplasty described by Moufarrege[12]. The most important feature of the technique was that the central U shaped pedicle was a total posterior pedicle. The "open sky" approach was used and all tissues were easily accessible. Thus, the desirable shape was given and maximum preservation of all anatomical structures was achieved. While central U shaped pedicle was being created, peripheral tissues were resected and posterior and superior connections of the pedicle were preserved completely. The pedicle directly carried the NAC and all vascular and neural connections of the pedicle were preserved.

### Surgical Technique

**Figure 3:**
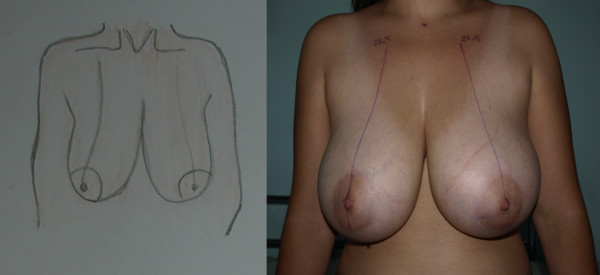
**Axis of the breast**.

**Figure 4:**
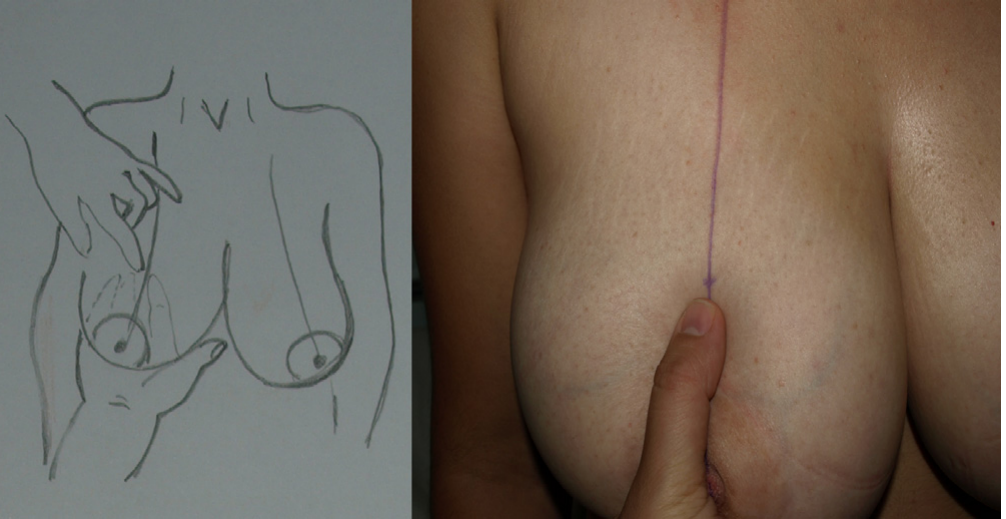
**Upper point of the keyhole pattern**.

**Figure 5:**
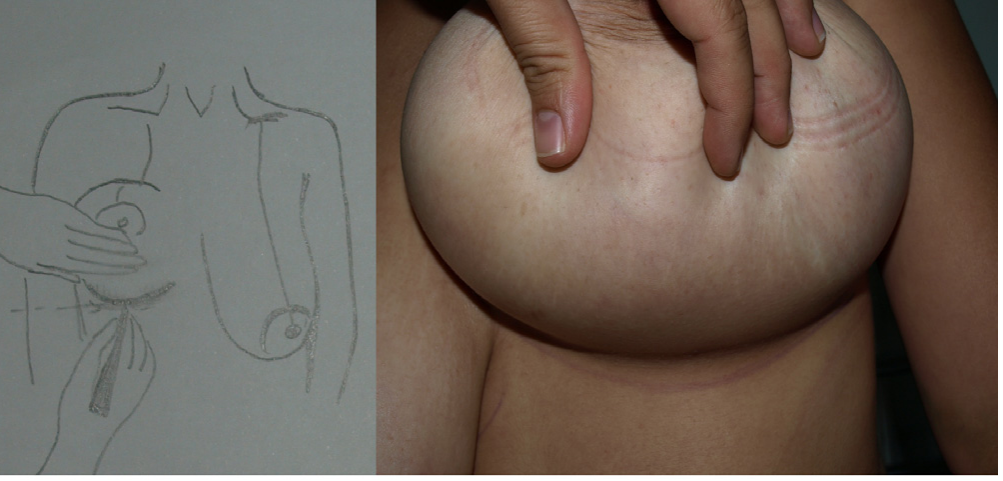
**Inframammarian fold marking**.

**Figure 6:**
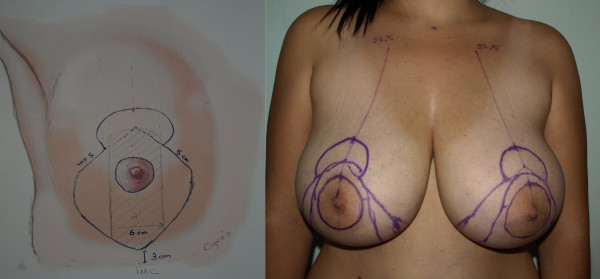
**Drawing of the keyhole pattern**.

**Figure 7:**
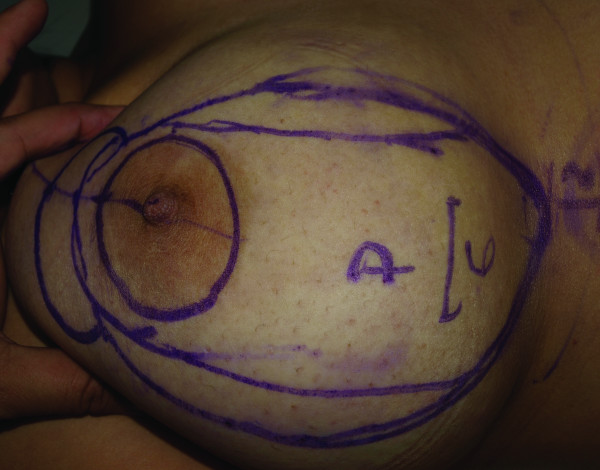
**Pre-operative markings of the patient on supine position**.

The first stage of the procedure was marking. A preoperative marking which was quite simple and easy to apply in all patients was developed. As Moufarrege described, the marking was performed when the patient was seated. In order to preserve the axis of each breast crossing the nipple, the vertical axis crossing the nipple and paralleling the margins of the breast was identified and this axis did not have to cross the mid-clavicular line (Figure 3). After the axis of the breast was detected, the inframammarian fold was marked and the upper point of the keyhole pattern was determined. This point was the place where the inframmamarian fold was located (Figure 4). Inframammarian fold was marked (Figure 5). Next, the standard keyhole pattern was marked. Extending arms of the pattern had an angle of 90 degrees and each was 5 cm in length (Figure 6). Moufarrege classified breasts into three based on their size when marking the standard keyhole pattern. We increased the angle between the arms of the keyhole to 135 degrees only in cases of gigantomastia. A larger angle is not more advantageous. In fact, creating a larger angle requires harvesting more skin and causes tension on the suture line, which may lead to difficulties in healing. Arms of the keyhole 5 cm in length formed a curve 3 cm above the inframammarian fold (Figure 7). Then, a vertical pedicle 6 cm in length running the midline of the breast was marked. It extended to 2 cm above the NAC in the superior part and till the end of the marked area in the inferior part. Last, the periareolar area 5 cm in diameter was marked.

**Figure 8:**
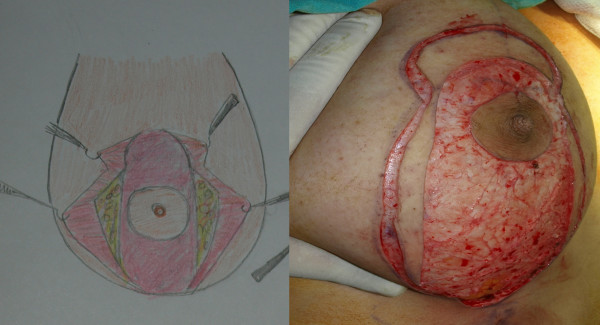
**De-epithelisation of the breast**.

**Figure 9:**
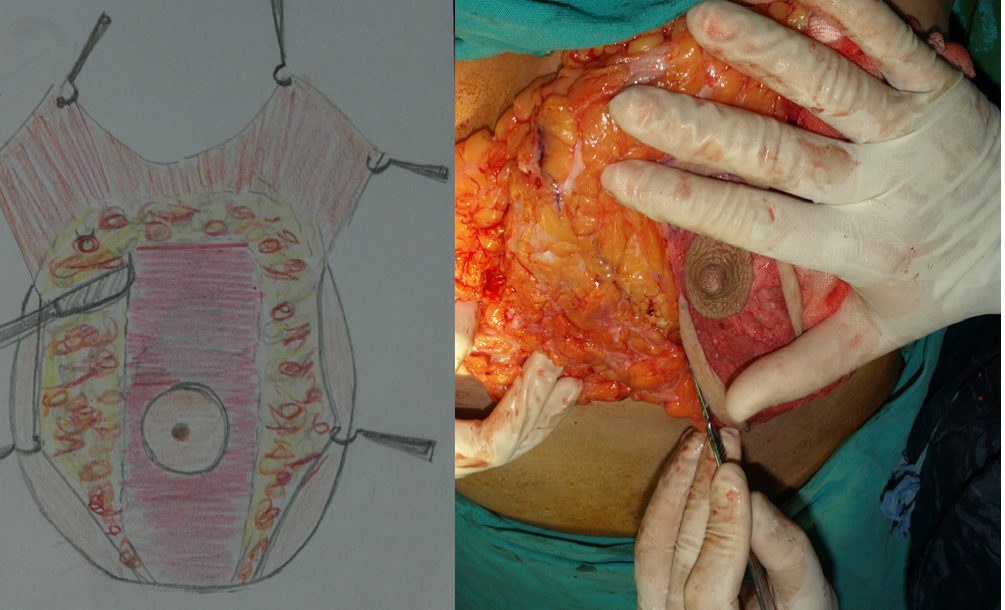
**Resection of medical and lateral tissues**.

**Figure 10:**
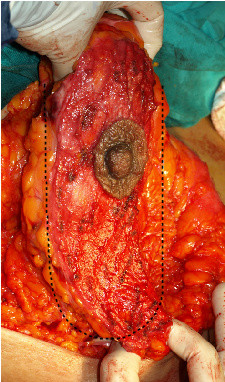
**U shaped total posterior pedicle 6 cm in width**.

**Figure 11:**
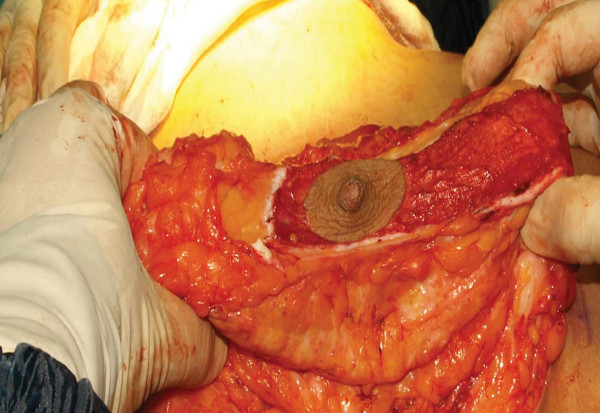
**Plication of the upper part of the pedicle**.

**Figure 12:**
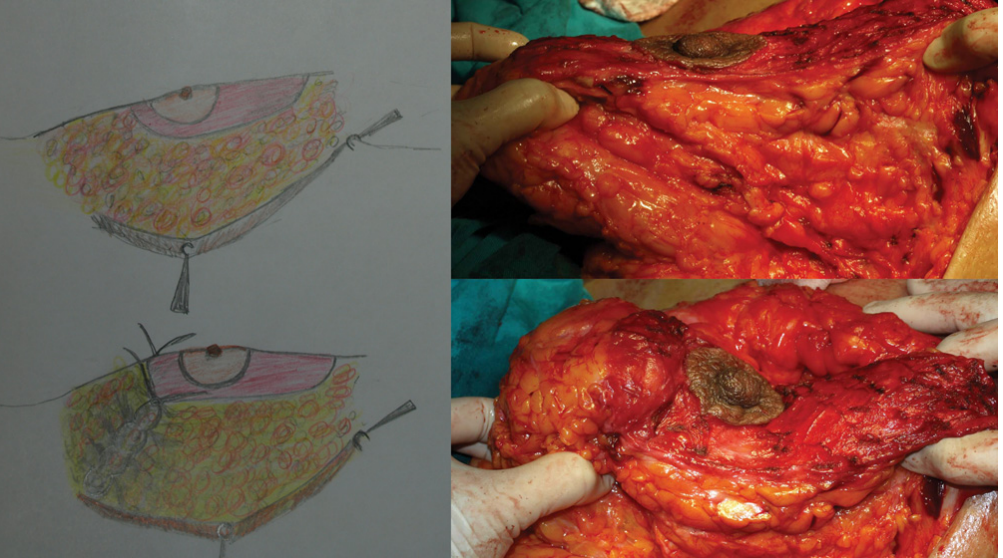
**View of the pedicle pre and post plicated**. Superior fullness was created with this plication.

The second stage was surgery. Patients were in the supine position with a slight flexion in the waist. The tumescent technique was used in all patients. After incisions appropriate for the markings were made, the skin on the pedicle was de-epithelized (Figure 8). Subsequently, skin flaps were undermined, starting in the medial. The breast including dermal fat was undermined from the gland to aponeurosis of the pectoralis major. At the end of undermining, the breast was completely exposed in the front view. Resection of the peripheral tissue started at the medial and continued at the lateral and at the inferior part minimally so as to create a 6 cm-U shaped pedicle in the middle (Figure 9). Resection margins in the inferior did not extend beyond the inframammarian fold and no resection was made in the superior. Unlike the posterior pedicle mammaplasty described by Moufarrege, the technique described here involved minimal resection in the inferior, which prevented excess in the horizontal part, and only one hole was created for drainage. Resection of the external quadrant extending to the subaxiallary region was performed gently and the areolar tissue in this area was preserved especially in cases of gigantomastia and extreme hypertrophy. After the resection was completed, a U shaped total posterior pedicle 6 cm in width remained in the middle (Figure 10). Following resection, conical plication was carried out to achieve superior fullness. Plication was performed in such a way to create a cone at the two o'clock and ten o'clock positions of the NAC with oblique continuous suture with 2/0 PDS (Figure 11 and 12) (Additional file 1). This plication is not dermal suspension, as seen in the video in additional file 1, areolar tissue, fat tissue and glandular tissue of breast are plicated. After conical plication was created, the breast was secured in its new position with temporary sutures running through inferior and superior parts of the NAC. One vertical suture was put 6 cm below the NAC and the area below this point was closed with pursing sutures. The subdermis was closed with 3/0 PDS, the vertical incision with 4/0 PDS and the periareolar region with 5/0 PDS without tension. One drainage tube was placed and temporary sutures were removed at the end of the operation (Additional file 2). Only a short vertical scar appeared in all cases and reverse T incision was avoided. Pressure dressing was done at the end of the operation and the drain tubes were removed within two days of the operations.

### Methodology

**Figure 13:**
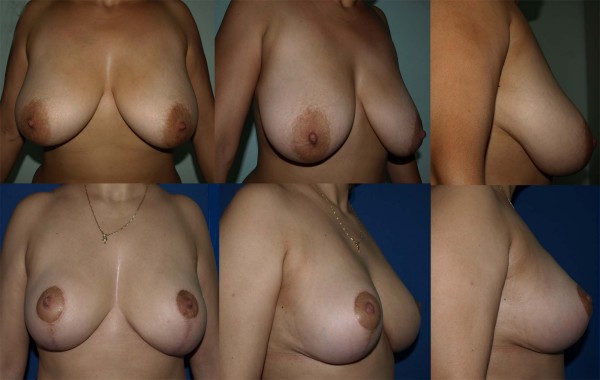
**Pre and post operative view of the patient operated with COPCUs mammaplasty for reduction of the breast**. 360 gr tissues were removed from each breasts.

**Figure 14:**
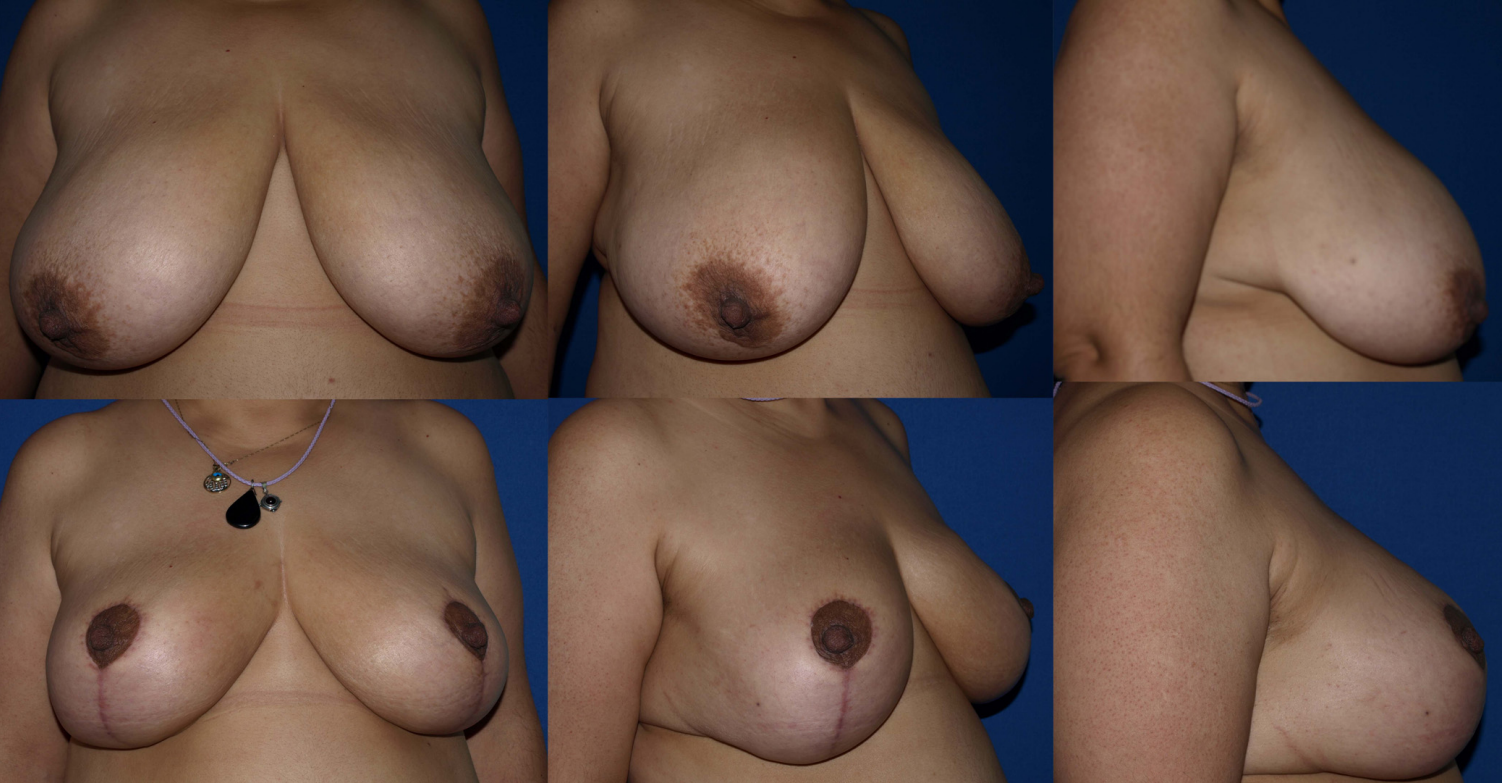
**Pre and post operative view of the patient with gigantomastia**. 1320 gr tissue were removed from each breasts.

**Figure 15:**
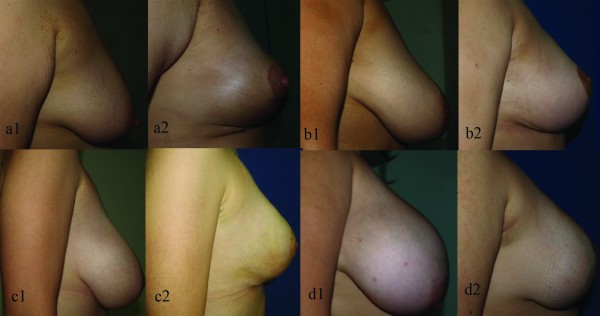
**Pre and postoperative lateral view of the patients a1-2: Mastopexy b1-2: Moderate breast hypertrophy c1-2: Marked breast hypertrophy d1-2: Gigantomastia**.

A detailed physical examination of the breast includes measurements of breast size, degree of ptosis, masses, superior pole fullness, nipple sternal distance, nipple-inframammarian fold distance were recorded. Anterior, lateral and two oblique photographs were taken to compare preoperative and postoperative superior fullness in controls of patients routinely (figure 13 and 14). Projections of the breasts were evaluated according to the lateral photographs of the patients (Figure 15). Superior fullness was evaluated with measurement of the breast projection. Also, weights of the resected tissue were recorded.

For quality scar evaluation we used visual analog scale. According to scale 0 was unacceptable scar must be corrected surgically and 10 was excellent scar. Patients were evaluated their scars after 6 months post-operatively. The patients were followed minimum six months post-operatively (6 months – 36 months).

Written informed consent was obtained from the patient for publication of this case report and accompanying images. A copy of the written consent is available for review by the Editor-in-Chief of this journal

## Results

Our technique was performed in 46 patients. The median age of the patients was 24 years, ranging from 17 years to 63 years. Data of patients are presented in Table 1. The median distance between the jugular notch to nipple was 31 cm, ranging between 22 cm and 48 cm. The mean resected tissue weight per breast was 564 gr, ranging from 273 gr to 1880 gr. Out of 46 patients included in the study, 38 underwent reduction mammaplasty (7 had gigantomastia), four mastopexy and four revision surgery. Out of four patients undergoing revision surgery, one had had inferior pedicle mammaplasty and three had had superior pedicle vertical scar mammaplasty before. All four patients had revision surgery for pseudoptosis.

**Table 1:** Data of the patients:

	Range	Average
Age	17–63	24 (median)

Jugular notch to nipple distance		

Pre-operative	22–48 cm.	31 cm. (median)

Post-operative	19–23 cm.	21 cm. (median)

Nipple to inframammarial crease		

Pre-operative	8–18 cm.	14.5 cm.

Post-operative	7–12 cm.	8 cm.

Breast projection		

Pre-operative	21–54 mm.	31 mm.

Post-operative	42–60 mm.	54 mm

Resection weight(per breast)	273–1880 gr	564 gr

Follow up	6–36 months	12 months

None of the patients had such major complications as necrosis, partial or total NAC loss. None of them required revision surgery. Two days after removal of the drainage tubes, two patients had minimal hematoma, which was treated conventionally. Two patients had about 1 cm opening on the NAC and suture line, but they healed spontaneously. Quality of the scars were scored by the patients themselves and median was 9, ranging with 7 to 10. All patients were satisfied with aesthetic results. We never performed scar revision surgery. Although we did not make investigational laboratory studies for the sensation of the nipple, none of the patients reported decreased sensual or sexual sensibility in the short term and long term. Six patients gave birth after operation and none of them had lactation difficulties according to their experience. According to their history, they did not need supplementation in first 6 months of breastfeeding.

## Discussion

Reduction mammaplasty is one of the most frequently performed operations by plastic surgeons. There have been numerous modifications of reduction mammaplasty [13]. It may be that the breast has a very complex geometric structure and very different anatomical components. Reduction mammaplasty techniques described so far are named after locations of pedicles. Among them are inferior, lateral, media, central, total posterior pedicle and mixed [14-18].

The leading cause of ongoing attempts to seek an ideal technique is complications such as failure to achieve the desirable aesthetic result, decreased or lack of lactation, decreased or loss of sensual and erogenous feeling of the nipple, insufficient projection and postoperative pseudoptosis and wound healing problems. Ultimate goal of any pedicle is to provide sufficient blood supply to the nipple areola complex[3]. It has been reported that superior pedicled mammaplasty causes considerable changes in blood circulation due to the transposition of the pedicle and that there is decreased NAC sensation in the superior pedicle in the short term. The nerves innervate the NAC can be easily injured with inferior pole resections with superior pedicle techniques [19,20]. Bottoming out, inferior pole excess or pseudo ptosis, is more frequent in inferior based pedicles [21]. Attempts to seek reduction mammaplasty techniques preserving the NAC emerged from the results of the studies by Bisenberger[22]. However, they revealed considerably high rates of complications. An effective way to prevent complications is to know the breast anatomy well, especially the vascular anatomy of the breast.

The classical pattern of blood supply to the breast was first described by Manchot in 1889 and later, Marcus showed that the breast had three different patterns of blood supply [23]. The most recent and extensive study on the issue was performed by Wuringer in 1998[6]. Wuringer examined 28 female breasts and described a horizontal septum extending from fifth rib toward the nipple, dividing the blood supply into a cranial and caudal network. Deventer on 27 cadaver dissections in 2004 demonstrated that the blood flow from the nipple had a quite different pattern and that each breast of the same person might have had differences in blood flow from the nipples[23]. These findings indicated that the tissue below the nipples, especially the horizontal septum should be preserved.

Nipple necrosis is the most frightening complication of reduction mammaplasty. The rates of nipple necrosis have been reported to be 2.1% in the superodermal pedicle[24], 2.3% in the superolateral[25] and 0.8% in inferior pedicle[26]. The leading cause of nipple necrosis is insufficient arterial blood supply or long-lasting venous congestion; this can be attributed to inadequate knowledge about the vascular anatomy of the NAC and use of long peripheral pedicle and the resultant distortion of the pedicle. However, theoretically total posterior pedicle described by Moufarrege and its modification COPCUs mammaplasty may avoid such complications.

Although the preoperative marking described in the present study resembles the bipedicle modification described by Khan in 2007, it differs in resection and pedicle considerably from that described by Khan[27]. It was inevitable that the horizontal diameter in bipedicled and total central pedicled techniques was larger than expected; fortunately, U shaped pedicle resolved this issue. In addition, two dead spaces -one in the medial and the other in the lateral- remain in bipedicled techniques and fluid and blood accumulate in these spaces in cases of insufficient drainage; however, in U shaped pedicle all spaces are connected to each other and one effective drainage tube allows collection of fluid and blood. Khan and Moufarrege reported that the biggest advantage of their techniques was preservation of blood supply to the nipple and easy mobilization of the breast towards the superior without loss of viability in the breast tissue. However, U shaped pedicle was more advantageous since it was connected to the NAC both in the superior and in the inferior.

At present, there is a general agreement that the most popular techniques are vertical scar mammaplasty and its modifications. Due to high rate of complications (especially wound healing, seroma) in vertical scar mammaplasty, many modifications were presented literature, but use of vertical scar technique for large breasts still is not widely accepted, especially with use of superior pedicle[28]. Rohrich et al presented results of a survey in 2004[29]. According to this report assessing the trends in breast reduction techniques among the members of the American Society for Aesthetic Plastic Surgery found that the most frequent complications for the limited incision technique group were suture spitting, the need for surgical revision and the loss of nipple sensation[28]. Advantage of the superior pedicle technique is an improved superior projection and a stable long-term shape of the breast as compared with the inferior pedicle techniques[30]. But the use of a superior pedicle supposedly increases the rate of areolar necrosis[28]. The techniques providing most effective blood supply to the breast are those with a central pedicle. The central pedicle technique in current use is the end result of serial modifications of Biesenberger's technique[22]. Balch[31] and Hester[32] used the classical T incision, Peixoto[33] and Hagerty [34] used vertical incision, Yousif[35] and Lalonde[36] used horizontal scar and Goes [37] used periareolar technique for central pedicle. Total posterior pedicle was described by Moufarrege[12,18]. It was called total posterior pedicle since the pedicle was just behind the NAC and the whole posterior pedicle was made of the gland. Total posterior pedicle achieved maximum gland and nipple security and Moufarrege reported low rates of complications in more than 10000 patients undergoing reduction mammaplasty. Moreover, none of them were major complications. Although Moufarrege technique was used of the severe gynecomastia[38], Moufarrege did not recommend total posterior pedicle for large breasts. The complications of the central breast reduction techniques are few and most of these are related to the inverted T-shsped scar, which is often unsatisfactory from the aesthetic point of view[39]. Since we used vertical scar, we did not have scar problem and never revision surgery was performed for the scar.

The management of gigantomastia is still debatable. Many authors propose that nipple areola graft can be utilized for the management of gigantomastia. In the present study, none of the patients with gigantomastia required grafting. Location of the NAC on a fully preserved posterior pedicle obviated the use of grafts.

It is of great importance to maintain the breast shape for a long time after reduction mammaplasty. However, gravity and tissue dynamics make it difficult. Recurrent ptosis may be a problem in all breast reduction techniques. Hammock technique[40], dermis strips[41], synthetic materials[42], fascia lata[43] and internal bra systems [44] have been used to eliminate recurrent ptosis. However, all techniques are based on the idea that the pedicle, like a suspensory ligament, should be suspended from the thorax wall. Around the areola, and especially below the areola, an area of de-epithelialised skin is preserved to be used as a bra-like support of the gland[45]. Dermis suspension gives a well-defined shape intra-operatively, which does not change significantly with time.

The philosophy is completely different in the technique presented here. De-epithelized area interacts with the above skin and thus helps to preserve conical plication and decreases effects of gravity. As far as we know, conical plication has not been described in the literature before. The conical plication which we developed is directed towards preservation of the juvenile breast look and superior fullness in the long term.

In 1985, Pennington performed plication and pedicle suspension in the pectoral fascia to prevent bottoming out, a frequently encountered complication of inferior pedicle, and reported his 20-year experience[11]. Pennington made plication, both superficial and deep, in the inferior pole. According to report plication had the effect of lifting the inframmary crease, narrowing the remaining breast mound, and creating a distinct fold in the pedicle. Unlike the plication by Pennington, plication in our technique is performed in the superior only to create a conical appearance. The suture technique used in our mammaplasty is similar to that described by Toonard for MACS lift[46]. The techniques which buries a lower pole breast parenchymal flap underneath a bipedicled pectoralis major muscle flap have not been supported worldwide because they make breast cancer screening difficult and they violate tissue compartment[47]. However, the technique presented here does not damage the tissue since it only involves plication and no problems due to plication were shown in postoperative mammography in the long term. Hawtof et al. studied 268 patients and concluded that complications were significantly more prevalent in women undergoing reductions of greater than 700 gr per breast[48]. The size of the breast was not associated with complications in the present series. This can be ascribed to safety of the pedicle. We did not observe breast feeding difficulties in our patients after surgery. Because peripheral reduction of the gland does not discontinue the lactiferous ducts and no disturbance of breast feeding is to be expected after this kind of reduction mammaplasty.

It is still debatable whether reduction mammaplasty can be used for revision surgery. In this study, four patients underwent our technique for revision. These patients had undergone inferior pedicle and vertical scar mammaoplasty before. The results showed that COPCUs mammaplasty could be used with success regardless of the pedicle created in prior operations.

The rate of complications in this study was found to be 9.5%. They were minor complications which did not require surgical treatment. The rate of complications in the literature has been found to range between 3% and 45% [49].

According to the results our experience, advantages of COPCUs mammaplasty provides complete safety of the NAC, creates the most natural breast in terms of tissue consistency and mobility, provides fullness in the superior part of the breast and excellent projection Our technique is very easy to perform and teach since open sky approach is used. It does not increase operation time and does not require liposuction. Reverse T scar is avoided and a very small vertical scar, which can be tolerated by patients, is created.

There are not any marked disadvantages of the technique. However, thinning likely to occur in elevation of dermal pedicles may cause skin problems. Although the patients included in this study were heavy smokers, they did not have skin loss. This indicates that dermal flaps have a rich blood supply.

Advantages of this technique should be proven with detailed investigational laboratory studies such as sensorial, angiographical and ductal screening tests. But according to our experience, we speculate that our technique can be used safely for breast reduction and mastopexy.

## Competing interests

The author declares that they have no competing interests.

## Authors' contributions

EC designed the study, performed all operations and prepared the manuscript.

## Supplementary Material

Additional file 1 **Plication of the breast**. The video file provided represents the plication of the upper part of the breast for youthful projection. Click here for file

Additional file 2 **COPCUs mammaplasty**. The video file provided represents the COPCUs mammaplasty in all steps and also pre and post operative views. Click here for file
